# Fat grafting associated with negative pressure wound therapy^1^

**DOI:** 10.1590/s0102-865020190090000007

**Published:** 2019-11-28

**Authors:** Gustavo Moreira Costa de Souza, Camila Camargos Bizzotto Amorim, Cristian Esteban Astudillo Vallejo, Marcelo Back Sternick, Sergio Moreira da Costa, Christiane Steponavicius Sobral, Lydia Masako Ferreira

**Affiliations:** I Fellow Master degree, Postgraduate Program in Translational Surgery, Division of Plastic Surgery, Department of Surgery, Universidade Federal de São Paulo (UNIFESP), Brazil. Design of the study, acquisition and analysis of data, manuscript writing; II MD, Resident, Fellow of Surgery, Postgraduate Program in Plastic Surgery, Plastic Surgery and Burn Clinic, Hospital Felício Rocho, Belo Horizonte-MG, Brazil. Acquisition of data; III MD, Orthopaedic Surgery Division, Hospital Felício Rocho, Belo Horizonte-MG, Brazil. Acquisition of data, manuscript writing; IV Head, Division of Plastic Surgery, Hospital Felício Rocho, Belo Horizonte-MG, Brazil. Design of the study, critical revision; V PhD, Plastic Surgeon, Division of Plastic Surgery, Hospital dos Defeitos da Face-Cruz Vermelha Brasileira, Sao Paulo-SP, Brazil. Critical revision; VI Head, Full Professor, Division of Plastic Surgery, UNIFESP, Researcher 1A-CNPq, Director Medicine III-CAPES, Sao Paulo-SP, Brazil. Critical revision; CNPq, Sao Paulo, SP, Brazil; CAPES, Sao Paulo, SP, Brazil

**Keywords:** Negative-Pressure Wound Therapy, Wound Healing, Transplantation, Autologous, Biocompatible Materials

## Abstract

**Purpose:**

To describe a case report of FG associated with NPWT in the treatment of complex wound on the distal third of the lower limb with bone exposure.

**Case Report:**

A 59-year-old patient with chronic left tibial osteomyelitis since childhood underwent extensive debridement of the distal tibial diaphysis (40% of bone thickness per 10 cm extension) and placement of bioactive glass S53P4. Distal necrosis occurred in the fasciocutaneous flap used as the primary bone coverage. After flap debridement, the case was resolved with FG, directly on the exposed bone and biomaterial, associated with NPWT. Three weeks after the first FG session over bony tissue, 100% granulation was achieved with NPWT. The closure was completed with thin laminated skin graft over the granulated wound area.

**Discussion:**

The association of FG and NPWT is not known in the clinical practice. Except for the only one experimental study described by Kao *et al*.^4^, the theme was not addressed in the medical literature before. In this clinical case, the result obtained regarding the granulation tissue formation drew attention and prevented the use of more complex flaps such as the microsurgical ones. Accelerated granulation tissue formation was observed, filling an extensive and deep bone defect, even with infected bone and biomaterial. Low morbidity and no complications were observed with the use of FG associated with NPWT. When the grafted fat was compacted with the NPWT, it seemed to behave as a true autologous biological matrix with large amount of cells. To date, scientific studies on fat grafting have focused on the cellular aspect (adipocytes and mesenchymal cells), growth factors and fat differentiation in different tissues. The property of aspirated adipose tissue as a biological matrix seemed to be revealed by the application of NPWT in association with FG. This new roll for the aspirated fat tissue may represent a new research field in plastic surgery.

## Introduction

Complex wounds represent a fundamental and prevalent issue in plastic surgery. In the 21^st^ century, new options of treatment have come up: negative pressure wound therapy (NPWT), fat grafting (FG) and biological matrices^1^ ^,^ ^2^. The objective of this previous note is to communicate the first clinical use of FG associated with NPWT for the treatment of a complex wound in the inferior left limb with exposed bone.

**Figure 1:**
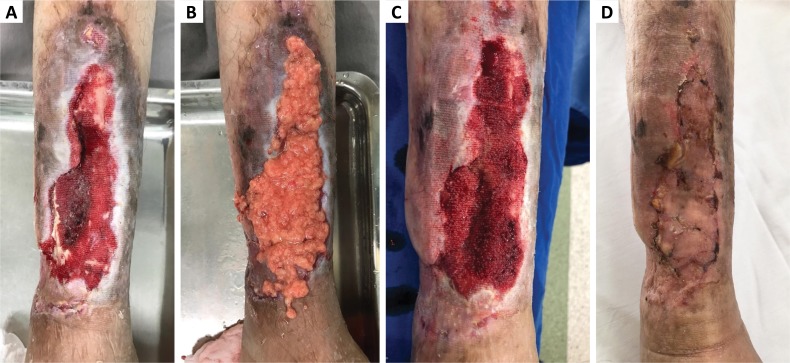
Sequence of the wound healing during treatment FG + NPWT. **A.** Complex wound with bone, bioactive glass and bone marrow exposure, missing 10cm of anterior tibial cortical bone, and infection after fasciocutaneous flap partial necrosis and debridement. **B.** FG + NPWT. **C.** Complete granulation tissue formation over the wound after two sessions of fat graft associated with negative pressure wound treatment. **D.** Skin graft.

In 04/18/2019, a 59 years old male patient, with chronic osteomyelitis in the left leg since childhood, was treated with distal tibial bone partial debridement (40% loss of bone in a 10cm segment) and application of bioactive glass S53P4 (BAG S53P4)^3^. The posterior medial fasciocutaneous flap, used for primary closure of the surgical wound, failed partially, with necrosis. After debridement of the flap, the infected wound with exposed bone and biomaterial was treated with FG associated with NPWT. After three weeks of treatment, granulation tissue covered all the bone and the BAG S53P4, making skin grafting possible for complete wound healing (Fig. 1).

The association of both methods (FG and NPWT) is new in clinical application. There is only one experimental research on this procedure, described by Kao *et al*.^4^. In this present case, a very fast granulation tissue formation was noticed, which prevented the utilization of a more complex flap, as the microsurgical ones. It was observed 100% granulation tissue formation over the hole exposed bone, even in the presence of BAG S53P4 and infection. Very low morbidity and no complications were noted with this treatment (FG associated with NPWT). When the grafted fat was pulled against the wound surface, due to the negative pressure therapy, it seemed to be transformed into an autologous biological matrix with large number of mesenchymal cells and adipocytes. The roll of the adipose tissue as a biological matrix can represent a new theme for research in the field of Plastic Surgery.
